# Investigating the Association Between Extended Participation in Collision Sports and Fluid Biomarkers Among Masters Athletes

**DOI:** 10.1089/neur.2023.0086

**Published:** 2024-01-30

**Authors:** Lauren P. Giesler, William T. O'Brien, Georgia F. Symons, Sabrina Salberg, Gershon Spitz, Robb Wesselingh, Terence J. O'Brien, Richelle Mychasiuk, Sandy R. Shultz, Stuart J. McDonald

**Affiliations:** ^1^ Department of Neuroscience, Central Clinical School, Monash University, Melbourne, Victoria, Australia.; ^2^ Turner Institute for Brain and Mental Health, Monash University, Melbourne, Victoria, Australia.; ^3^ Department of Neurology, Alfred Hospital, Melbourne, Victoria, Australia.; ^4^ Department of Medicine, Royal Melbourne Hospital, The University of Melbourne, Parkville, Victoria, Australia.; ^5^ Health Sciences, Vancouver Island University, Nanaimo, British Columbia, Canada.

**Keywords:** concussion, GFAP, inflammation, neurodegeneration, NfL, p-tau-181, tau, telomere, UCH-L1

## Abstract

Traumatic brain injuries (TBIs) and concussions are prevalent in collision sports, and there is evidence that levels of exposure to such sports may increase the risk of neurological abnormalities. Elevated levels of fluid-based biomarkers have been observed after concussions or among athletes with a history of participating in collision sports, and certain biomarkers exhibit sensitivity toward neurodegeneration. This study investigated a cohort of 28 male amateur athletes competing in “Masters” competitions for persons >35 years of age. The primary objective of this study was to compare the levels of blood and saliva biomarkers associated with brain injury, inflammation, aging, and neurodegeneration between athletes with an extensive history of collision sport participation (i.e., median = 27 years; interquartile range = 18–44, minimum = 8) and those with no history. Plasma proteins associated with neural damage and neurodegeneration were measured using Simoa^®^ assays, and saliva was analyzed for markers associated with inflammation and telomere length using quantitative real-time polymerase chain reaction. There were no significant differences between collision and non-collision sport athletes for plasma levels of glial fibrillary acidic protein, neurofilament light, ubiquitin C-terminal hydrolase L1, tau, tau phosphorylated at threonine 181, and brain-derived neurotrophic factor. Moreover, salivary levels of genes associated with inflammation and telomere length were similar between groups. There were no significant differences between groups in symptom frequency or severity on the Sport Concussion Assessment Tool–5th Edition. Overall, these findings provide preliminary evidence that biomarkers associated with neural tissue damage, neurodegeneration, and inflammation may not exhibit significant alterations in asymptomatic amateur athletes with an extensive history of amateur collision sport participation.

## Introduction

Repetitive traumatic brain injuries (TBIs) and concussions are common in collision sports, such as the football codes, and combat and equestrian sports.^1–3^ Considerable research has focused on the potential effects of concussion and TBI exposure in current or retired athletes with lengthy careers at the college or professional sports level.^4,5^ However, collision sports are also commonly participated in at the community level, and, in some cases, athletes continue to partake in these sports into middle age. In Australia, for example, “Masters” competitions for persons >35 years of age are common for collision sports, including Australian football. Although the health benefits of sport participation are numerous, it is important to understand how prolonged amateur/community collision sport participation into older years may influence neurological health. Previous research has extensively focused on professionals, retired professionals, and young athletes, leaving the Masters cohort notably under-represented.

Fluid biomarkers can potentially serve as indirect indicators of neurological injury or disease.^6^ Proteins such as glial fibrillary acidic protein (GFAP), neurofilament light (NfL), ubiquitin C-terminal hydrolase L1 (UCH-L1), tau, and tau phosphorylated at threonine 181 (p-tau-181) have been shown to be elevated in blood after TBIs^7,8^ or in persons with age-related neurodegeneration or neurodegenerative disease.^9^ Evidence suggests that fluid biomarkers associated with inflammation may be elevated in collision sports athletes,^10,11^ and chronic neuroinflammation has been implicated in cognitive decline and neurodegeneration.^12^ Brain-derived neurotrophic factor (BDNF) is a neurotrophin with important roles in the development and maintenance of the central nervous system, with alterations in BDNF messenger RNA (mRNA) and protein levels found in studies of brain injury and neurodegenerative disease.^13,14^ In addition, shortened saliva telomere length has been associated with aging processes and neurodegeneration,^15^ as well collision sport participation in cohorts of younger athletes.^16^ Therefore, panels consisting of these promising biomarker candidates hold the potential to offer insights into the neurological well-being of older, highly active athletes who have been extensively involved in collision sports.

This pilot study aims to compare blood and salivary biomarkers between older Australian football Masters athletes with an extensive history of collision sport participation compared with non-collision sport field hockey Masters athletes.

## Methods

### Participants

A total of 32 male Masters athletes were recruited for this study through convenience sampling, including 19 active collision sport (i.e., Australian football) athletes from an amateur Australian football club, and 13 active non-collision sport (i.e. field hockey) athletes from a field hockey club. Inclusion criteria for the collision sport cohort were: 1) age >35 years; 2) significant history of collision sport participation, defined here as >8 years. Non-collision controls were >35 years of age, had participated in non-collision sport for >8 years, and had no history of collision sport participation. For sporting history classification, collision sport participation was defined as any participation in Australian football, gridiron, rugby, ice hockey, lacrosse, or combat sport. Sports outside of this classification were ruled to be non-collision. This research was approved by the Alfred Health Human Research Ethics Committee (#187/18).

### Clinical questionnaires

Participants completed questionnaires pertaining to demographics, and medical, sporting, and drug and alcohol history. The Sport Concussion Assessment Tool–5th Edition (SCAT5) was used to quantify concussion-related symptoms.

### Blood analysis

Venous blood was collected into ethylenediaminetetraacetic acid tubes, with plasma isolated after centrifugation at 1100*g* and stored at −80°C. A Simoa HD-X Analyzer was used to quantify GFAP, NfL, UCH-L1, and tau (Neurology 4-plex B), pTau-181 (pTau-181 V2 Advantage), and BDNF (BDNF Discovery kit) as per the manufacturer's instructions. Assays were performed in a temperature-controlled laboratory by an experimenter blinded to the clinical information. All samples were tested in duplicate and measured above the lower limit of quantification for GFAP (9.38 pg/mL), NfL (0.500 pg/mL), tau (0.125 pg/mL), p-tau-181 (0.085 pg/mL), and BDNF (0.029 pg/mL). For UCH-L1, 10 of 28 samples were below the lower limit of detection and thus assigned this value: 2.43 pg/mL. The average interplate coefficient of variation of samples for GFAP, NfL, UCH-L1, tau, p-tau-181, and BDNF was 9%, 7%, 46%, 6%, 6%, and 5%, respectively.

### Saliva analysis

Saliva samples, collected using Oragene DNA (OG-500) kits per the manufacturer's instructions for future RNA and DNA isolation, underwent RNA extraction with the Allprep DNA/RNA Mini Kit. RNA concentration and quality were assessed using the Nanodrop 2000. Two micrograms of RNA were reverse transcribed into complementary DNA (cDNA) using qScript™ XLT cDNA SuperMix. For qRT-PCR, 10 ng of cDNA, 1X SYBR Green FastMix ROX, and 0.5-μM forward and reverse primers were used, with all samples run in duplicate on a 96-well plate using the CFX Connect-Real-Time PCR Detection system. Analysis utilized the 2^(-ΔΔCT) method, normalized against housekeeping genes Cyca and Ywhaz, following Pfaffl^17^ and Bonefeld.^18^

For telomeres, DNA extraction utilized the QIAamp DNA Mini Kit per the manufacturer's instructions, with DNA concentration and quality evaluated by the Nanodrop 2000. DNA was diluted to 20 ng/μL with TE buffer for downstream quantitative real-time polymerase chain reaction (qRT-PCR). Samples were run in duplicate on the CFX Connect-Real-Time PCR Detection system with 1X SYBR Green FastMix ROX and appropriate primers, totaling 20 μL per well. Relative telomere quantification involved comparing the telomere to single-copy 36B4 gene ratio (T/S), calculated as approximately [2Ct(telomere)/2Ct(36B4)]−1 = −2−ΔCt. Telomere length was determined using the linear regression equation: y = 1910.5x + 4157, with y representing telomere length and x corresponding to −2−ΔCt.^19^ Primer sequences and cycling parameters for qRT-PCR analysis are available upon request.

### Statistical analysis

Statistical analyses were conducted in R software (version 4.0.3; R Foundation for Statistical Analysis, Vienna, Austria). All analyses were two-tailed, with a significance level of *p* < 0.05. Demographic variables were compared using unpaired *t*-tests. Nominal variables were evaluated using Pearson's chi-squared (χ^2^) test. Biomarker data underwent natural logarithm transformation before analysis. To investigate the relationship between sport group and fluid biomarkers, we performed multiple linear regression analysis controlling for age and body mass index (BMI).

## Results

### Demographic information

Four participants initially recruited to the study were excluded from all analyses; 1 collision athlete had not played collision sport for >8 years, and 3 non-collision athletes reported a significant history of collision sport participation (i.e., ≥20 years). Two additional participants were unable to complete the saliva collection, but remained in the study for all other analyses. Demographics, sporting, and medical histories of each group are detailed in Table 1. Primary differences in cohorts included were that collision athletes had more years of collision sport participation compared to the non-collision sport group (*p* < 0.001). There were no differences in participant age, years of sport participation, participant age at the commencement of sport, or years since most recent concussion between groups. However, the collision sport group reported having sustained more concussions than those in the non-collision sport group (*p* = 0.040).

**Table 1.** Participant Demographics of Non-Collision and Collision Sport Athletes

	Non-collision (*n* = 10)	Collision (n = 18)	*p *value
Age	52.0 (47.8–56.1)	54.0 (48.7–60.3)	0.601
BMI	26.0 (24.7–27.7)	26.7 (25.3–28.1)	0.471
Years of sport participation	40.0 (23.5–45.5)	41.0 (20.0–46.0)	0.918
Years of collision sport participation	0.0 (0.0–0.0)	27.0 (17.8–44.3)	**<0.001**
Age commenced sport	7.0 (5.5–16.3)	8.0 (6.0–9.0)	0.173
Age commenced collision sport	N/A	9.5 (9.0–13.0)	
Consecutive years playing collision sport before study			**<0.001**
N/A-Miss	100 (100.0%)	3 (16.7%)	
0–5 years	0 (0.0%)	3 (16.7%)	
6–10 years	0 (0.0%)	1 (5.6%)	
>10 years	0 (0.0%)	11 (61.1%)	
History of concussion			0.092
N/A-Miss	1 (10.0%)	0 (0.0%)	
Yes	4 (40.0%)	14 (77.8%)	
No	5 (50.0%)	4 (22.2%)	
No. of past concussions	0.0 (0.0–1.0)	2.5 (0.8–7.0)	**0.040**
Medical Dx	0.0 (0.0–0.0)	0.0 (0.0–2.3)	0.244
Self-report	1.0 (1.0–1.8)	3.0 (1.8–6.8)	0.195
History of LOC			0.123
N/A-Miss	6 (60.0%)	4 (22.2%)	
Yes	2 (20.0%)	9 (50.0%)	
No	2 (20.0%)	5 (27.8%)	
No. of LOCs	0.0 (0.0–1.5)	1.0 (0.0–1.3)	0.477
Years since last concussion	15.0 (3.5–30.3)	6.0 (2.0–21.5)	0.540
Hypertension			0.927
Yes	1 (10.0%)	2 (11.1%)	
No	9 (90.0%)	16 (88.9%)	
Diabetes mellitus			0.662
Yes	1 (10.0%)	1 (5.6%)	
No	9 (90.0%)	17 (94.4%)	
Heart conditions			0.410
Yes	3 (30.0%)	3 (16.7%)	
No	7 (70.0%)	15 (83.3%)	
Lung conditions			0.236
Yes	2 (20.0%)	1 (5.6%)	
No	8 (80.0%)	17 (94.4%)	
Thyroid disease			0.172
Yes	1 (10.0%)	0 (0.0%)	
No	9 (90.0%)	18 (100%)	
Renal insufficiency			0.172
Yes	1 (10.0%)	0 (0.0%)	
No	9 (90.0%)	18 (100%)	
Stroke			0.448
Yes	0 (0.0%)	1 (5.6%)	
No	10 (100.0%)	17 (94.4%)	
Headache/migraine			0.507
Yes	4 (40.0%)	5 (27.8%)	
No	6 (60.0%)	13 (72.2%)	
Seizure/epilepsy			0.662
Yes	1 (10.0%)	1 (5.6%)	
No	9 (90.0%)	17 (94.4%)	
Cancer			0.629
Yes	1 (10.0%)	3 (16.7%)	
No	9 (90.0%)	15 (83.3%)	
Arthritis			0.373
N/A-Miss	0 (0.0%)	1 (10.0%)	
Yes	1 (10.0%)	5 (27.8%)	
No	9 (90.0%)	12 (66.7%)	
Psychiatric illness			**0.039**
Yes	0 (0.0%)	6 (33.3%)	
No	10 (100.0%)	12 (66.7%)	

No significant differences in demographic variables were observed, with the exception of years of collision sport participation, consecutive years playing collision sport before study, number of past concussions, and history of psychiatric illness. Results are presented as median (IQR) or frequency (proportion).

BMI, body mass index; N/A, not applicable; Dx, diagnosis; LOC, loss of consciousness; IQR, interquartile range.

### Symptomatology

There were no differences in self-reported symptom number (*p* = 0.347) or self-reported symptom severity (*p* = 0.324) on the SCAT5 between groups.

### Plasma biomarkers

**FIG. 1.**
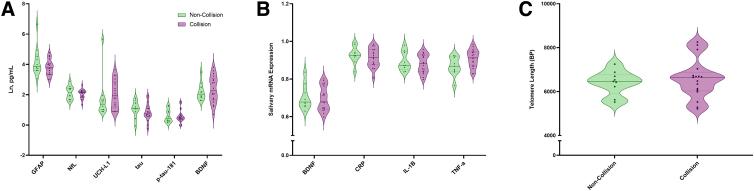
Fluid biomarkers in collision and non-collision sport Masters athletes. (**A**) No differences in plasma concentrations of GFAP, NfL, UCH-L1, tau, p-tau-181, or BDNF were observed between collision and non-collision Masters athletes. (**B**) No differences in saliva biomarkers BDNF, CRP, IL-1β, or TNF-α were observed between collision and non-collision Masters athletes. (**C**) No differences in telomere length were observed between collision and non-collision Masters athletes. BDNF, brain-derived neurotrophic factor; CRP, C-reactive protein; GFAP, glial fibrillary acidic protein; IL-1β, interleukin-1 beta; NfL, neurofilament light; p-tau-181, tau phosphorylated at threonine 181; TNF-α, tumor necrosis factor-alpha; UCH-L1, ubiquitin C-terminal hydrolase L1.

As shown in Figure 1A, there were no effects of collision sport participation for plasma levels of GFAP (estimate = −0.43, confidence interval [CI] = −1.02 to 0.17, *p* = 0.153), NfL (estimate = −0.09, CI = −0.34 to 0.17, *p* = 0.482), UCH-L1 (estimate = 0.29, CI = −0.67 to 1.25, *p* = 0.541), tau (estimate = −0.20, CI = −0.66 to 0.25, *p* = 0.368), p-tau-181 (estimate = −0.05, CI = −0.39 to 0.30, *p* = 0.782), and BDNF (estimate = 0.07, CI = −0.54 to 0.67, *p* = 0.817).

### Salivary messenger RNA

For BDNF (estimate = −0.01, CI = −0.08 to 0.07, *p* = 0.873), C-reactive protein (CRP; estimate = −0.02, CI = −0.06 to 0.03, *p* = 0.488), interleukin-1 beta (IL-1β; estimate = −0.01, CI = −0.06 to 0.04, *p* = 0.593), and tumor necrosis factor alpha (TNF-α; estimate = 0.05, CI = 0.00–0.10, *p* = 0.062), no effects of collision sport were found (Fig. 1B).

### Telomere length

For telomere length (Fig. 1C), no effect of collision sport was observed (estimate = 0.02, CI = −0.09 to 0.13, *p* = 0.708).

## Discussion

This pilot study found that an extended history of amateur collision sport participation into middle age did not have a significant effect on plasma biomarker levels of neuronal (i.e., UCH-L1), axonal (i.e., tau, p-tau-181, and NfL), or astroglial pathology (i.e., GFAP). Moreover, we found no differences in measures of plasma BDNF or salivary mRNA and telomere measures. Our results are consistent with Major and colleagues, in which no association was found between remote history of concussion and serum biomarker levels (i.e., GFAP, UCH-L1, NfL, tau, and p-tau-181) in younger adult amateur Australian football players.^19^ Similarly, Swann and colleagues recently found no elevation in plasma GFAP, NfL, and tau in former athletes with an extensive history of concussion.^20^ However, our previous studies have demonstrated some significant findings regarding young adult Australian football players. Specifically, we observed elevated serum protein levels of tau and p-tau, as well as shorter telomere length, in comparison to non-collision sport controls.^16^ Further, we have previously found that male players with a history of concussion exhibited higher levels of serum IL-1β compared with male players without a concussion history, and a positive correlation between IL-18 levels and the number of years of collision sport participation.^10^

Although the sample size in the current study is relatively small, and biomarkers, biofluid type, and platforms used to analyze are not identical to the previous studies, the preliminary findings suggest a lack of biomarker changes in Masters Australian football players with an extensive history of collision sport participation.

Aside from athlete age and biomarker differences, there may be other notable distinctions between the current cohorts of Masters studies and previous research conducted on younger Australian football players participating in “Senior” adult football. First, Senior male football players in the aforementioned studies reported more recent concussion exposure (median ∼2.5 years) compared to the Masters collision sport athletes in this study (6 years). Second, there are some differences in the way in which Australian football is played between the young adult and Masters competitions, with a “stronger emphasis on eliminating all forms of rough contact” in Masters football.^21^ Player movement (e.g., speed) and behavioral attitudes (e.g., aggression) are likely different between levels, potentially influencing the magnitude and frequency of TBIs.^22^ Third, Masters football is played less frequently (i.e., fortnightly) than Senior football (weekly). Considered together, TBI exposure is likely reduced in Masters Australian football and may explain some discrepancies in findings to that in younger players.

Although Masters Australian football may involve reduced impact exposure compared to Senior football played by younger adults, it is worth noting that the majority of the current cohort also had a significant history of participation in this form of the game earlier in life. Comparable levels of well-studied plasma biomarkers, such as GFAP, NfL, and p-tau-181, between collision sports athletes and non-collision controls,^20^ as well as their consistency with existing literature on healthy adults,^23–25^ likely indicate a lack of significant active neurodegeneration in these athletes. However, it is important to recognize that fluid biomarkers provide an indirect “snapshot” of current neurodegeneration, and advanced neuroimaging techniques, such as diffusion tensor imaging, can offer direct indications of compromised microstructural integrity resulting from past damage or degeneration. Future studies should explore potential variations in brain microstructure between different cohorts of collision sport athletes and non-collision controls. Additionally, longitudinal biomarker evaluation and cognitive testing may yield valuable insights into the potential effects of extended collision sports participation.

In conclusion, this study presents preliminary evidence suggesting that a panel of fluid biomarkers associated with brain damage, neurodegeneration, and inflammation may not exhibit significant elevation in active older Australian football Masters athletes who have a history of extensive and ongoing participation in collision sports.

## Abbreviations Used

BDNF: brain-derived neurotrophic factor
BMI: body mass index
cDNA: complementary DNA
CI: confidence interval
CRP: C-reactive protein
GFAP: glial fibrillary acidic protein
IL-1β: interleukin-1 beta
mRNA: messenger RNA
NfL: neurofilament light
p-tau-181: tau phosphorylated at threonine 181
qRT-PCR: quantitative real-time polymerase chain reaction
SCAT5: Sport Concussion Assessment Tool–5th Edition
TBI: traumatic brain injury
TNF-α: tumor necrosis factor-alpha
UCH-L1: ubiquitin C-terminal hydrolase L1

## References

[1] Orchard J, Seward H, Orchard MJ. 2012 AFL injury report. Australian Football League: Docklands, Australia; 2013.

[2] Bernick C, Hansen T, Ng W, et al. Concussion occurrence and recognition in professional boxing and MMA matches: toward a concussion protocol in combat sports. Phys Sportsmed 2021;49(4):469–475; doi: 10.1080/00913847.2020.1856631 33251911

[3] Rueda MAF, Halley WL, Gilchrist MD. Fall and injury incidence rates of jockeys while racing in Ireland, France and Britain. Injury 2010;41(5):533–539; doi: 10.1016/j.injury.2009.05.009 19524903

[4] Casson IR, Viano DC, Powell JW, et al. Twelve years of National Football League concussion data. Sports Health 2010;2(6):471–483; doi: 10.1177/1941738110383963 23015977 PMC3438866

[5] Manley G, Gardner AJ, Schneider KJ, et al. A systematic review of potential long-term effects of sport-related concussion. Br J Sports Med 2017;51(12):969–977; doi: 10.1136/bjsports-2017-097791 28455362 PMC5466926

[6] Zetterberg H, Blennow K. Fluid biomarkers for mild traumatic brain injury and related conditions. Nat Rev Neurol 2016;12(10):563–574; doi: 10.1038/nrneurol.2016.127 27632903

[7] McCrea M, Broglio SP, McAllister TW, et al. Association of blood biomarkers with acute sport-related concussion in collegiate athletes: findings from the NCAA and Department of Defense CARE Consortium. JAMA Netw Open 2020;3(1):e1919771; doi: 10.1001/jamanetworkopen.2019.19771 31977061 PMC6991302

[8] McDonald SJ, O'Brien WT, Symons GF, et al. Prolonged elevation of serum neurofilament light after concussion in male Australian football players. Biomark Res 2021;9(1):4; doi: 10.1186/s40364-020-00256-7 33422120 PMC7797141

[9] Ashton NJ, Hye A, Rajkumar AP, et al. An update on blood-based biomarkers for non-Alzheimer neurodegenerative disorders. Nature Reviews Neurology 2020;16(5):265–284; doi: 10.1038/s41582-020-0348-0 32322100

[10] O'Brien WT, Symons GF, Bain J, et al. Elevated serum interleukin-1β levels in male, but not female, collision sport athletes with a concussion history. J Neurotrauma 2021;38(10):1350–1357; doi: 10.1089/neu.2020.7479 33308001

[11] Nitta ME, Savitz J, Nelson LD, et al. Acute elevation of serum inflammatory markers predicts symptom recovery after concussion. Neurology 2019;93(5):e497–e507; doi: 10.1212/WNL.0000000000007864 31270219 PMC6693429

[12] Faden AI, Loane DJ. Chronic neurodegeneration after traumatic brain injury: Alzheimer disease, chronic traumatic encephalopathy, or persistent neuroinflammation? Neurotherapeutics 2015;12(1):143–150; doi: 10.1007/s13311-014-0319-5 25421001 PMC4322076

[13] Wurzelmann M, Romeika J, Sun D. Therapeutic potential of brain-derived neurotrophic factor (BDNF) and a small molecular mimics of BDNF for traumatic brain injury. Neural Regen Res 2017;12(1):7–12; doi: 10.4103/1673-5374.198964 28250730 PMC5319242

[14] Phillips HS, Hains JM, Armanini M, et al. BDNF mRNA is decreased in the hippocampus of individuals with Alzheimer's disease. Neuron 1991;7(5):695–702; doi: 10.1016/0896-6273(91)90273-3 1742020

[15] Shay JW. Telomeres and aging. Curr Opin Cell Biol 2018;52:1–7; doi: 10.1016/j.ceb.2017.12.001 29253739

[16] Symons GF, Clough M, O'Brien WT, et al. Shortened telomeres and serum protein biomarker abnormalities in collision sport athletes regardless of concussion history and sex. J Concussion 2020; doi: 10.1177/2059700220975609

[17] Pfaffl MW. A new mathematical model for relative quantification in real-time RT–PCR. Nucleic Acids Res 2001;29(9):e45; doi: 10.1093/nar/29.9.e45 11328886 PMC55695

[18] Bonefeld BE, Elfving B, Wegener G. Reference genes for normalization: a study of rat brain tissue. Synapse 2008;62(4):302–309; doi: 10.1002/syn.20496 18241047

[19] Major BP, McDonald SJ, O'Brien WT, et al. Serum protein biomarker findings reflective of oxidative stress and vascular abnormalities in male, but not female, collision sport athletes. Front Neurol 2020;11:549624; doi: 10.3389/fneur.2020.549624 33117257 PMC7561422

[20] Swann OJ, Turner M, Heslegrave A, et al. Fluid biomarkers and risk of neurodegenerative disease in retired athletes with multiple concussions: results from the International Concussion and Head Injury Research Foundation Brain health in Retired athletes Study of Ageing and Impact-Related Neurodegenerative Disease (ICHIRF-BRAIN study). BMJ Open Sport Exerc Med 2022;8(3):e001327; doi: 10.1136/bmjsem-2022-001327 PMC9438045 36111130

[21] Australian Football League. What to Expect—fun safe inclusive footy for men & women over 25. AFL Masters: Spotswood, VIC, Australia; 2023. Available from: https://aflmasters.com.au/what-to-expect/ [Last accessed: July 1, 2023].

[22] Saw R, Finch CF, Samra D, et al. Injuries in Australian rules football: an overview of injury rates, patterns, and mechanisms across all levels of play. Sports Health 2018;10(3):208–216; doi: 10.1177/1941738117726070 28825878 PMC5958447

[23] Baiardi S, Quadalti C, Mammana A, et al. Diagnostic value of plasma p-tau181, NfL, and GFAP in a clinical setting cohort of prevalent neurodegenerative dementias. Alzheimers Res Ther 2022;14(1):153; doi: 10.1186/s13195-022-01093-6 36221099 PMC9555092

[24] Chen CH, Cheng YW, Chen YF, et al. Plasma neurofilament light chain and glial fibrillary acidic protein predict stroke in CADASIL. J Neuroinflammation 2020;17(1):124; doi: 10.1186/s12974-020-01813-5 32321529 PMC7175500

[25] Chouliaras L, Thomas A, Malpetti M, et al. Differential levels of plasma biomarkers of neurodegeneration in Lewy body dementia, Alzheimer's disease, frontotemporal dementia and progressive supranuclear palsy. J Neurol Neurosurg Psychiatry 2022;93(6):651–658; doi: 10.1136/jnnp-2021-327788 35078917 PMC9148982

